# Characterization of Endophytic *Streptomyces griseorubens* MPT42 and Assessment of Antimicrobial Synergistic Interactions of Its Extract and Essential Oil from Host Plant *Litsea cubeba*

**DOI:** 10.3390/antibiotics8040197

**Published:** 2019-10-28

**Authors:** Quang Huy Nguyen, Hai Van Nguyen, Thi Hanh-Nguyen Vu, Son Chu-Ky, Thu Trang Vu, Ha Hoang, Ngoc Tung Quach, Thi Lien Bui, Hoang Ha Chu, Thi Nhan Khieu, Samira Sarter, Wen-Jun Li, Quyet-Tien Phi

**Affiliations:** 1 Vietnam Academy of Science and Technology (VAST), Graduate University of Science and Technology (GUST), 18 Hoang Quoc Viet, Cau Giay, Hanoi 10000, Vietnam; huynguyen.biotech@gmail.com (Q.H.N.); chuhoangha@ibt.ac.vn (H.H.C.); 2 Vietnam Academy of Science and Technology (VAST), University of Science and Technology of Hanoi (USTH), 18 Hoang Quoc Viet, Cau Giay, Hanoi 10000, Vietnam; 3 Institute of Biotechnology, Vietnam Academy of Science and Technology, 18 Hoang Quoc Viet, Cau Giay, Hanoi 10000, Vietnam; hagiangyeu@yahoo.com (T.H.-N.V.); hoanghapcb@ibt.ac.vn (H.H.); quachngoctung88@gmail.com (N.T.Q.); builien1521995@gmail.com (T.L.B.); 4 School of Biotechnology and Food Technology, Hanoi University of Science and Technology, 1, Dai Co Viet, Hai Ba Trung, Hanoi 10000, Vietnam; nguyenhaivan.181@gmail.com (H.V.N.); thutrangvu1981@yahoo.com (T.T.V.); 5 Ministry of Education and Training, 49 Dai Co Viet, Hai Ba Trung, Hanoi 10000, Vietnam; ktnhan@moet.gov.vn; 6 CIRAD, UMR ISEM, University Montpellier, F-34398 Montpellier, France; samira.sarter@cirad.fr; 7 ISEM, Université de Montpellier, CNRS, EPHE, IRD, F-34398 Montpellier, France; 8 School of Life Sciences, Sun Yat-Sen University, Guangzhou 510275, China; liact@hotmail.com; 9 Yunnan Institute of Microbiology, Yunnan University, Kunming 650091, China

**Keywords:** antimicrobial activity, endophytic actinomycete, essential oil, medicinal plants, membrane permeability, *Litsea cubeba*, synergistic effect and *Streptomyces griseorubens*

## Abstract

The present study aimed to evaluate the synergistic effects of the crude ethyl acetate extract (CEAE) from endophytic actinomycete MPT42 and essential oil (EO) of the same host plant *Litsea cubeba*. The isolate MPT42, exhibiting broad-spectrum antimicrobial activities and harboring all three antibiotic-related biosynthetic genes *pks*-I, *pks*-II, and *nrps*, was identified as *Streptomycete griseorubens* based on an analysis of the morphology, physiology, and 16S rDNA sequence. Minimum inhibitory concentrations (MICs) and the fractional inhibitory concentration index were used to estimate the synergistic effects of various combined ratios between CEAE or antibiotics (erythromycin, vancomycin) and EO toward 13 microbial strains including pathogens. *L. cubeba* fruit EO, showing the main chemical constituents of 36.0% citral, 29.6% carveol, and 20.5% limonene, revealed an active-low against tested microbes (MICs ≥ 600 μg/mL). The CEAE of *S. griseorubens* culture exhibited moderate–strong antimicrobial activities against microbes (MICs = 80–600 μg/mL). Analysis of the mechanism of action of EO on *Escherichia coli* ATCC 25922 found that bacterial cells were dead after 7 h of the EO treatment at 1 MIC (5.5 mg/mL), where 62% cells were permeabilized after 2 h and 3% of them were filament (length ≥ 6 μm). Combinations of CEAE, erythromycin, or vancomycin with EO led to significant synergistic antimicrobial effects against microbes with 4–16 fold reduction in MIC values when compared to their single use. Interestingly, the vancomycin–EO combinations exhibited a strong synergistic effect against five Gram-negative bacterial species. This could assume that the synergy was possibly due to increasing the cell membrane permeability by the EO acting on the bacterial cells, which allows the uptake and diffusion of antimicrobial substances inside the cell easily. These findings in the present study therefore propose a possible alternative to combat the emergence of multidrug-resistant microbes in veterinary and clinics.

## 1. Introduction

The misuse and overuse of antibiotics for human and animal health management has become a main factor contributing to the rapid emergence and dissemination of antibiotic-resistant bacteria, which poses a serious threat to global public health [1]. Consequently, antibiotic resistance increases the cost of healthcare, the risk of treatment failure, and the fatality rate. Thus, the findings of novel antimicrobial agents and alternative therapeutic strategies are urgently needed [2]. Medicinal plants are rich sources of essential oils (EOs) and secondary metabolite compounds that are often used as natural and safe medicines in the treatment of infectious diseases. Additionally, the combination of antimicrobial agents (EO and antibiotics) may lead to synergistic effects against pathogens that can reduce the side effects of antibiotics, enhance the bioavailability, lower the therapeutic dose, and minimize the antimicrobial resistance [3]. 

The *Litsea* genus is one of the most diverse genera of evergreen trees or shrubs in the *Lauraceae* family, comprising roughly 400 species over the world [4]. The *Litsea cubeba* species is frequently found in southern China, Japan, Taiwan, and South-East Asia including Vietnam, and is widely and safely used in cosmetics, and traditional medicine for the treatment of headache, fatigue, muscle pain, depression, sores and furuncles [5,6,7]. Interestingly, *L. cubeba* EO exhibits considerable bioactivities such as antibacterial [8], antifungal [9], antioxidant [10], and anticancer activities [11]. Furthermore, rich-EO-content medicinal plants like *L. cubeba* and *Cinnamomum* spp. could also be one of the prevalent sources for antibiotic-producing endophytic actinobacteria conveying novel features [12,13]. In fact, it has been previously demonstrated that the ecology and evolution of endophytic microorganisms might be affected by the chemical compositions of host plant EO and the participation of endophytes in the biotransformation of EO [14]. 

Our previous study primarily explored the accelerated antibacterial effects of the cell-free supernatant of an isolated endophytic actinomycete and *L. cubeba* EO [8]. Nevertheless, further study providing additional insights into the synergistic interactions between endophyte-derived secondary metabolites and EO from host medicinal plants should be addressed, which may generate a potential strategy to combat the emergence and spread of multidrug-resistant pathogens. Therefore, the present study aimed to characterize the broad-spectrum antibacterial producing endophyte *Streptomyces griseorubens* MPT42 associated with *L. cubeba* and evaluate the combinatory antimicrobial activities of the crude ethyl acetate extract (CEAE) of the culture broth of *S. griseorubens* MPT42 and fruit EO of the host plant against various pathogens including multidrug-resistant bacteria. In addition, the effects of the *L. cubeba* EO on the bacterial cell viability and morphology of a model *Escherichia coli* were also investigated to reveal the possible mode of action for the combination of CEAE and EO. Although vancomycin seems to have no effect on Gram-negative bacteria, a recent study showed that the combination of vancomycin with other antibiotics displayed a strong synergic effect against Gram-negative bacteria [15]. Therefore, the present study examined the potential synergistic interactions of EO with erythromycin (a broad-spectrum antibacterial substance) or vancomycin (a Gram-positive antibacterial substance) against various Gram-positive and negative pathogens.

## 2. Results

### 2.1. Identification and Characterization of Antibiotic-Producing Endophytic Actinomycete MPT42

In order to screen the antibacterial activity of isolates, 35 out of 143 endophytic actinomycetes potentially producing antibiotics were isolated from different organs of *L. cubeba* (Lour.) Pers plants [16]. Among them, the strain MPT42 isolated from the stem of the host plant inhibited the growth of the majority of tested bacteria with inhibitory zones ranging from 11.4 to 44.0 mm (Table 1). 

**Figure 1:**
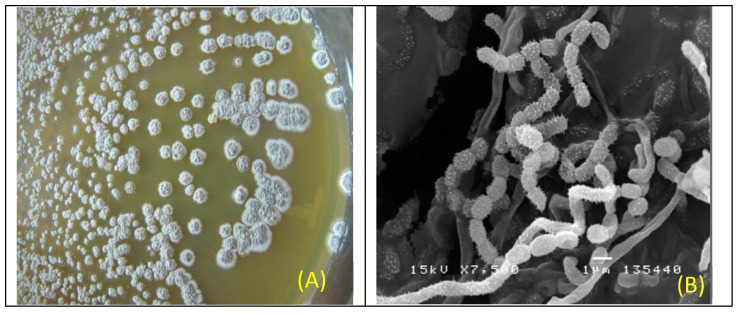
Colony morphology (**A**) and scanning electron microscope showing the spore-chain morphology and spore-surface ornamentation of the endophytic actinomycete strain MPT42 grown on ISP2 agar medium for 14 days at 28 °C at a magnification of 7500× (**B**).

On ISP2 agar medium, aerial mycelium color of the MPT42 colonies was changed from white to gray after five days of cultivation (Figure 1A) and none of the pigments was observed until 30 days. The spore chain consisted of oval-shaped spores with spiral and spiny surfaces (Figure 1B).

The physiological characteristics of strain MPT42 are described in the Table 2, which exhibited well-aerobic growth on an ISP2 medium at pH 8.0, temperature of 35 °C, and NaCl concentration of 1%. The strain MPT42 can utilize different 11 carbon and 10 nitrogen sources (Table 2). In addition, the PCR results revealed that endophytic actinomycete MPT42 possessed three secondary metabolite biosynthetic genes: *pks*-I, *pks*-II, and *nrps* (Table 2).

**Figure 2:**
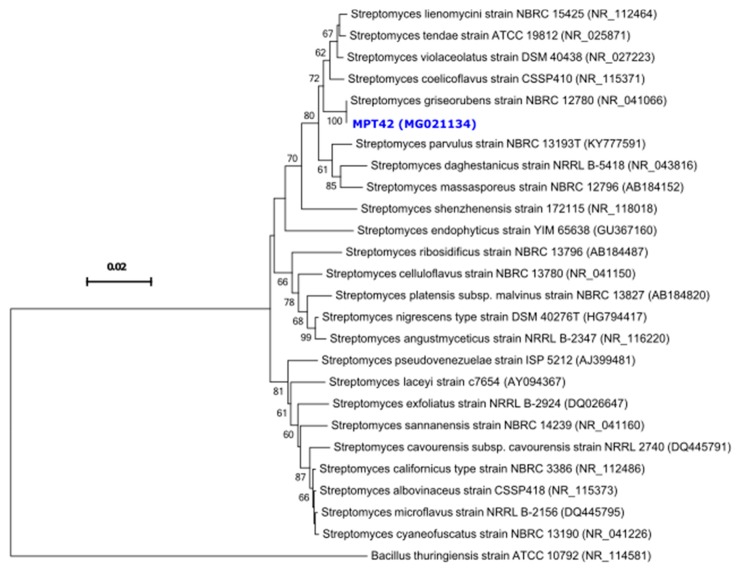
Neighbor-joining phylogenetic tree based on 16S rRNA gene sequences of *Streptomyces griseorubens* MPT42 and type strains retrieved from GenBank (accession numbers are shown in parentheses). *Bacillus thuringinensis* strain ATCC 10792 was used as the outgroup. Only bootstrap values >50% are shown.

Analysis of the 16S rRNA gene sequence identified the strain MPT42 as a member of the *Streptomyces* genus (homology of 99.5–100%). The neighbor-joining phylogenetic tree indicated the close relationship between strain MPT42 and related *Streptomyces* species and showed the highest homology to *Streptomyces griseorubens* type strain NBRC 12780 (bootstrap value of 100%, Figure 2), therefore, it was assigned as *Streptomyces griseorubens* MPT42.

### 2.2. Antimicrobial Activity of the Crude Ethyl Acetate Extract (CEAE) and Essential Oil (EO)

The CEAE of *S. griseorubens* MPT42 inhibited the growth of 13 tested microbes at different levels (Table 3). Briefly, it was highly active (MICs ≤ 100 μg/mL) against *S. aureus*, *E. coli,* and *S.* Typhimurium, while the MICs were moderate (between 200 and 600 μg/mL) toward *B. subtilis*, *A. hydrophila, V. parahaemolyticus, B. cereus, P. aeruginosa, L. innocua,* and *P. vulgaris* [17]. For methicillin-resistant *Staphylococcus epidermidis* ATCC 35984 (MRSE) and methicillin-resistant *Staphylococcus aureus* ATCC 33591 (MRSA), the MICs were 0.4 and 1.5 mg/mL, respectively.

Analysis of the chemical constituents by gas chromatography-mass spectrometry showed that the main compositions of 98%-purity EO were citral (36.0%), carveol (29.6%), and limonene (20.5%). Interestingly, the antimicrobial activity of the individual substances has been reported previously [9,18]. Here, we examined the antimicrobial activity of the total EO constituents to explore potential synergistic effects. Nevertheless, in concordance with a previous study [9,19], the *L. cubeba* EO showed low MICs (≥ 700 μg/mL) against the tested microbes (Table 3) [17].

### 2.3. Effect of the L. cubeba EO on Viability and Cell Morphology of E. coli 

**Figure 3:**
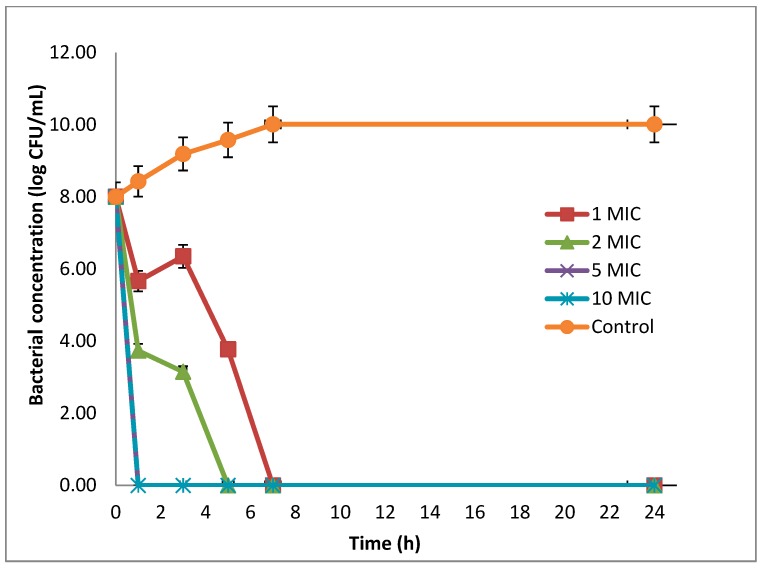
Time–kill kinetics of the *L. cubeba* fruit EO on the viability of *E. coli* ATCC 25922 (n = 3 ± SD). The bacterial cells were treated with different concentrations of EO (1, 2, 5, and 10 MIC) for 24 h. The control was EO-untreated cells.

Time-killing assays showed that the viability of *E. coli* ATCC 25922 cells was gradually reduced according to the increase in EO concentration (Figure 3), where the living cells were not detected after 5–7 h of incubation at EO concentrations of 2 and 1 MIC, respectively (MIC = 5.5 mg/mL). In the EO-free wells, the concentration of living cells was dramatically increased (reaching 10 log_10_ CFU/mL after 7 h). In contrast, over 4.2 log_10_ CFU/mL (50% reduction) of the cell viability compared with the initial cell concentration was obtained at 2 MIC (3.74 log_10_ CFU/mL) and 1 MIC (3.77 log_10_ CFU/mL) of the EO concentrations after 1 and 5 h, respectively.

**Figure 4:**
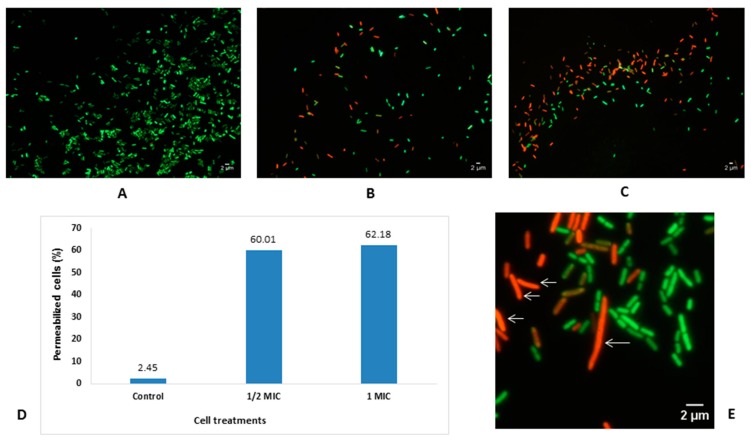
Effects of *L. cubeba* fruit EO on the viability of *E. coli* ATCC 25922. Fluorescence microscopic images with the LIVE/DEAD Baclight kit of *E. coli* cells after 2 h of exposure to the EO. Control: EO-untreated cells (**A**); EO-treated cells at 0.5 MIC (**B**) and at 1 MIC (**C**), and proportion of permeabilized cells (**D**). Viable cells are indicated by green fluorescence, whereas cells with damaged membranes are indicated by red fluorescence. The white arrows indicate elongated cells (**E**) and the filament cells were >6 μm in length. Scale bar represents 2 μm.

Cell viability evaluated by using the LIVE/DEAD BacLight viability kit revealed that the number of intact cells decreased significantly in the culture treated with the EO (0.5 and 1 MIC) compared to the control (Figure 4A–C). Moreover, proportions of the membrane-damaged cells were over 62% in the treated samples, whereas this proportion was only 2.5% in the control (Figure 4D).

The morphology and size changes of the *E. coli* cells were examined on images of EO-treated and EO-untreated cells (Figure 4E). The length of the bacterial cells exposed to EO dramatically increased 1.3–1.4 fold (corresponding to 2.41 and 2.54 µm, respectively) when compared with the untreated cells (1.86 μm). Notably, the proportion of filament cells (length ≥ 6 μm) was 3% in the 1 MIC EO-treated *E. coli* populations. None of the elongated or filament cells was observed in the EO-untreated bacterial populations.

### 2.4. Combined Antimicrobial Effects Against Microbial Strains

To enhance antimicrobial potencies, the combinations of CEAE with EO were tested against the 12 microbial species including several pathogens (Table 4). The vast majority of the combinations significantly decreased MIC from 4 to 16 folds when compared with the use of CEAE and EO separately. Specifically, the combinations of CEAE-EO led to synergistic antimicrobial interactions (*n* = 8) against *B. cereus*, *B. subtilis*, *S. aureus*, MRSE, *L. innocua*, *A. hydrophila*, *E. *coli**, and *P. aeruginosa*. An additive effect (*n* = 3) was observed toward MRSA, *Proteus vulgaris*, and *Vibrio parahaemolyticus* (Table 4). The highest antimicrobial synergy was found for the combination of CEAE–EO against *L. innocua* and MRSE (FIC = 0.19) (Table 4).

In the present study, the combinations of EO with either antibiotics E or VA also increased the antimicrobial effects toward the microbial species tested. Almost all of these antibiotic–EO combinations led to a decrease of 2–16 MIC folds when compared with the single use of E or VA. Specifically, the combinations of E with EO also led to seven synergistic and four additive effects, while the VA–EO combinations resulted to 10 synergistic—additive effects (Table 4). It is worth noting that the VA–EO combinations exhibited three synergistic antimicrobial effects on Gram-negative bacteria *A.s hydrophila* and *P. aeruginosa* (FICIs of 0.31 and 0.33, respectively), and additive effects toward *E. coli, P. vulgaris* and *V. parahaemolyticus* (FICIs between 0.54 and 0.56).

## 3. Discussion

So far, *S. griseorubens* species has mainly been found in soils [12,22,23] and was previously demonstrated as a strong antagonistic species conveying broad antifungal activities [24]. The present research revealed for the first time the activity of the endophytic actinomycete *S. griseorubens* MPT42 isolated from the stem of *L. cubeba.* Its CEAE extract exhibited a broad-spectrum antimicrobial activity toward various microbial types including drug-sensitive and multidrug-resistant pathogens. It showed moderate—strong inhibitory effects with MICs values between 80–600 µg/mL on 10 different microbes. Since many novel antibiotics exhibiting strong effects against multiple-drug resistant bacteria have been isolated from medicinal plant-associated endophytic *Streptomyces* spp. in the last decades [22,25,26,27,28], endophytic *S. griseorubens* MP42 could be a potential candidate for the production of valuable bioactive substances.

In agreement with previous reports [9,18,29], the present study demonstrated the potential growth inhibiting effect of *L. cubeba* EO (MICs of 0.70–5.5 mg/mL) toward a broad array of microbial species, compared with EOs derived from other medicinal plants. For instance, *Ocimum gratissimum* EO inhibited both Gram-positive bacteria (*S. aureus* and *Bacillus* spp.) at the concentration of 93.7–150 mg/mL and Gram-negative bacteria (*E. coli, P. aeruginosa, S*. Typhimurium, *Klebsiella pneumoniae, Proteus mirabilis*) at the concentration of 107–750 mg/mL [30]. Another study showed that *Cinnamomum* EO (Lauraceae family) possessed low MIC values ranging from 1.2 to 12.5 mg/mL against *E. coli*, *C. jejuni*, *S. aureus*, *S. enteritidis, S.* Typhimurium, *L. innocua,* and *L. monocytogenes* [31,32]. Thus, different chemical constituents in the EO of medicinal plant species affect their potential antimicrobial activity. Using *E. coli* as a model for exploring the mechanism of action of *L. cubeba* fruit EO, we demonstrated that EO-treated cells were rapidly killed after 2 h of treatment at the EO concentrations ≥ 2 MIC. It is worth noting that the cells showed extraordinary deformation and elongation. Our study found citral, carveol, and limonene as the main constituents, accounting together for 86.1% of total *L. cubeba* fruit EO, concordant with previous studies [9,18]. Since the effect of each substance on microbes was examined separately in these studies, therefore, the present study aimed at investigating the antimicrobial inhibitory effects possessed by the total EO. This combination showed broad-spectrum inhibitory effects against various microbial species tested. In fact, citral, carveol, and limonene are phenolic substances with polarity characteristics that might act on the bacterial cell membrane, inactivate cell adhesions, and/or interact with the outer and inner membrane proteins [19,29,33,34]. Consequently, the EO treatment might result in the weakening of cell structure and inhibition of cell growth by creating holes on the cell wall and increasing the cell permeability [29]. 

Since the modes of action of EO and antibiotics on microorganisms are different, the combination of both agents could enhance antimicrobial effects. The present study also found that combinations of EO with the CEAE of *S. griseorubens* MP42 or with either E or VA significantly reduced the MIC values on almost all microbes tested in comparison with the individual effects (MIC reductions of 4–16 folds and 2–16 folds for CEAE and antibiotics, respectively), therefore showing significant synergistic antimicrobial effects. Thus, the synergistic effects of *S. griseorubens* MPT42- extract and *L. cubeba* fruit EO might be due to the fact that EO disintegrates the bacterial cell membrane, influences the cell wall structure, forming holes and gaps in the cell wall [3,29,35,36]. These processes make antibiotic-like substances diffusive inside bacterial cells, where they might then inhibit metabolic processes, protein and/or DNA synthesis, etc., leading to cell death [3,29,37]. Although combinations of different types or generations of antibiotics have shown synergistic effects [38,39], the use of an appropriate EO in combination with antibiotics against bacterial pathogens could bring advantageous alternatives to reducing the use of conventional antibiotics as well as the emergence of multidrug-resistant bacteria [40]. For instance, streptomycin and kanamycin combined with lemongrass (*Cymbopogon citratus*) EO exhibited synergistic or additive effect against *S.* Typhimurium [37]. The combination of gentamicin or tobramycin with tea tree (*Melaleuca alternifolia*) EO had a synergistic effect against both *E. coli* and *S. aureus* [37]. Notably, VA combined with Shiraz oregano (*Zataria multiflora*) EO showed synergistic activity against MRSA [35]. It is well known that Gram-negative bacteria are normally resistant to VA as this antibiotic cannot significantly penetrate the outer membrane of cells. In the present study, the use of VA was not as the control for the inhibitory growth of Gram-negative bacteria, but aimed to discover the potential synergistic antimicrobial effects of VA–EO against Gram-negative bacteria. We found that in the presence of EO, the MICs of VA toward Gram-negative bacteria were significantly reduced and had strong synergistic inhibitory effects for *A. hydrophila* and *P. aeruginosa*, and additive effects on *E. *coli,* P. vulgaris*, and *V. parahaemolyticus*. This finding could be explained though two mechanisms: first, the EO could increase the permeability of the cell wall that allows the VA diffusion inside cells; second, interactions between VA and other antimicrobial substances of EO lead to synergistic antimicrobial effects. In fact, the drug interaction network possesses a special property, and in many cases, the antibiotics interact purely synergistically, leading to increased antibacterial activity [41]. A study showed that the combination of VA with either nitrofurantoin or trimethoprim had a strong synergic effect against *E. coli* at concentrations of VA as low as 6.25 μg/mL [15]. The result of our study demonstrated that the EO–VA combination increased antimicrobial inhibitory activities against Gram-negative bacteria when compared with the individual effect of VA. Altogether, our study underlines that the combination of antimicrobial substances with the EO of medicinal plants brings greater and broader effects toward various pathogens and that the synergy can lead to multi-targeted effects on the inhibition of microbial growth and viability.

## 4. Materials and Methods 

### 4.1. Screening for Antibacterial Activity 

The antibacterial activity of endophytic actinomycete MPT42 was examined by using the agar diffusion method [42] against Gram-negative bacteria (*Escherichia coli* ATCC 25922, *Proteus vulgaris* ATCC 49132, *Pseudomonas aeruginosa* ATCC 9027, *Salmonella* Typhimurium ATCC 14028, and *Enterobacter aerogenes* ATCC 13048) and Gram-positive bacteria (*Listeria innocua* ATCC 33090, *Bacillus cereus* ATCC 13061, *Bacillus subtilis* ATCC 11778, *Staphylococcus aureus* ATCC 25923, and Methicillin-Resistant *Staphylococcus epidermidis* ATCC 35984 (MRSE). 

### 4.2. Characteristics of the Endophytic Actinomycete MPT42

The strain MPT42 was cultured at 28 °C on International Streptomyces Project 2 (ISP2) agar medium for 14 days to characterize the biophysical-biochemical properties [43,44]. The spore chain morphology and spore ornamentation were analyzed by scanning electron microscope (SEM) JSM-5410 (JEOL, Tokyo, Japan) at a magnification of 7500x. The effects of different conditions (ranges of pH, NaCl concentration and temperature, carbon and nitrogen sources) to the growth were investigated as previously described [45].

### 4.3. Amplification of Secondary Metabolite Biosynthetic Genes and 16S rRNA Encoding Gene

The amplification of the 16S rRNA gene sequence of actinomycete MPT42 was performed by using the universal primer pair 27F (5′-TAACACATGCAAGTCGAACG-3′) and 1429R (5′-GGTGTGACGGGCGGTGTGTA-3′) [46]. The multiple sequence alignment was employed for the MPT42 and type strains (sequences retrieved from GenBank, National Center for Biotechnology Information (NCBI)) by using MEGA6 [47]. The phylogenetic tree was computed by using the neighbor-joining method with 1000 bootstrap in MEGA6 [47] and *Bacillus thuringiensis* strain ATCC 10792 (NR_114581) was used as the outgroup branch. The 16S rRNA gene sequence of strain MPT42 was deposited at GenBank (NCBI) under the accession number MG021134.

The presence of genes non-ribosomal polyketide synthases (*nrps*)*,* polyketide synthase type I (*pks*-I), and type II (*pks*-II) encoding for secondary metabolite biosynthesis in actinomycetes was explored by using three sets of degenerated primer pairs A3F (5’-GCS TAC SYS ATS TAC ACS TCS GG-3’) and A7R (5’-SAS GTC VCC SGT SCG GTA S-3’), K1F (5’-TSA AGT CSA ACA TCG GBC A-3’) and M6R (5’-CGC AGG TTS CSG TAC CAG TA-3’), KSaF (5’-TSG CST GCT TGG AYG CSA TC-3’), and KSaR (5’-TGG AAN CCG CCG AAB CCG CT-3’), respectively [48,49]. PCR components and conditions were performed as previously described [50].

### 4.4. Preparation of L. cubeba Fruit EO and CEAE From MPT42 Culture

The *L. cubeba* fruit EO were extracted by hydro-distillation using a Clevenger-type apparatus for 4 h, and then dried over anhydrous sodium sulfate. Finally, the EO density obtained was 0.88 g/mL (98% purity) and it was stored at 4 °C in a dark bottle until use. The *L. cubeba* fruit EO was then completely dissolved in distilled water supplemented with 0.5% Tween 80 (an emulsifier usually used to dissolve EOs in water) to achieve final concentrations ranging from 0.25 mg/mL to 64 mg/mL.

The strain MPT42 was cultivated in YIM38 antibiotic-producing medium at 28 °C with 200 rpm shaking. After five days, the culture broth was centrifuged and the cell-free supernatant (CFS) was extracted with ethyl acetate (1:1, v/v) and evaporated to determine the weight of CEAE. After that, the CEAE solution was prepared in ethanol with the concentration ranging from 0.0625 to 16 mg/mL.

### 4.5. Determination of Minimum Inhibitory Concentration

The minimum inhibitory concentrations (MICs) of the CEAE and EO were determined separately by using serial microdilution assays [7] against 12 bacterial species: *E. coli*, *P. vulgaris*, *P. aeruginosa*, *S.* Typhimurium, *L. innocua*, *B. cereus*, *B. subtilis*, *S. aureus*, MRSE, *Aeromonas hydrophila* ATCC 35654, *Vibrio parahaemolyticus* ATCC 17802, and methicillin-resistant *Staphylococcus aureus* (MRSA) ATCC 33591.

For each microorganism, suspensions were prepared in Mueller Hilton broth (MHB, Merck) to obtain the final concentration of 10^7^ CFU/mL. Each well contained 20 μL of the CEAE or EO and 180 μL of each bacterial suspension. After incubation 24 h at 30 °C for *A. hydrophila* and *V. parahaemolyticus* and at 37 °C for the other strains, the optical density (OD) was measured at 600 nm using a microplate reader (Bio-Rad Model 680, Japan). All experiments were performed in triplicate. The MIC value was determined as the lowest concentration showing the inhibition of bacterial growth. The standard antibiotics erythromycin (E) and vancomycin (VA) were used to evaluate the synergetic antimicrobial effects of the EO–antibiotic combinations by a microdilution checkerboard assay (described below). The antibiotic concentrations were prepared in a range from 0.25 to 512 μg/mL, and the test was performed under the same conditions.

### 4.6. Time-Killing Assay

In order to investigate the activity of the *L. cubeba* EO against pathogenic bacteria, the time-kill curve assay was performed using *E. coli* ATCC 25922 as a model [19]. The experiments were designed as follows: tubes including bacterial suspension (10^8^ CFU/mL) were exposed to four different EO concentrations (1, 2, 5, and 10 MIC) and control tube (bacterial culture without EO). The tubes were incubated at 37 °C for 24 h with stirring at 120 rpm/min. Total viable bacteria were enumerated by spreading 100 µL of culture on Muller Hinton agar plates and after 0, 1, 3, 5, 7, and 24 h of incubation at 37 °C, bacterial colonies were counted and the bacterial concentration was expressed as log_10_ CFU/mL. Assays were carried out in triplicate.

### 4.7. Effects of L. cubeba EO on Bacterial Cell Viability and Morphology

The effect of the *L. cubeba* fruit EO on cell viability was evaluated using the LIVE/DEAD BacLight viability kit (Invitrogen^TM^, Molecular Probes Inc., OR, USA) [19]. Briefly, the *E. coli* cultures were prepared in MHB as described above, then each tube containing 5 mL of cell suspension was incubated with 1 MIC and 0.5 MIC of the EO at 37 °C. After 2 h of exposure, the cells were harvested by centrifugation at 10,000x g for 10 min, then the supernatant was removed and pellets were re-suspended in NaCl 0.85%. The mixture of SYTO 9 and propidium iodide (PI) was added into the suspension with the ratio of 1:1. The cell suspension with 0.5% Tween 80 (v/v) and without EO was used as the control. After 15 min of staining, cells were examined under the microscope on 1% agarose (in water)-covered slides. Using a LEICA DM6000 photomicroscope equipped with an ORCA-ER C4742-80 camera (Hamamatsu, Japan), at least 20 photomicrographs were taken of different fields of view as previously described [51]. The percentages of cells stained with PI (permeabilized cells) and SYTO 9 (viable cells) were determined. 

The effect of EO on the size of bacterial cells was evaluated by analyzing fluorescence microscopy images with ImageJ [19]. *E. coli* cell length was measured and recorded with and without the EO treatment. Cell length was measured as the distance along the two axes of the cell. The dividing cells that were not separated yet were counted and measured as one single cell. Cells were counted as two separated cells only when the constriction was completed. At least 1000 cell sizes were measured for each experiment. Filament cells were considered when the cell length was ≥6 μm.

### 4.8. Microdilution Checkerboard Assays

The checkerboard method was performed using 96-well microtiter plates to obtain the fractional inhibitory concentration (FIC) index for determining the interaction between the CEAE and EO; and those between EO and antibiotics (E and VA) [21]. Serial two-fold dilutions were prepared to examine the combinatory antimicrobial activity of the CEAE and EO (25 pair combined concentrations from 0.0625 MIC to 1 MIC). For each well, 20 μL CEAE and 20 μL EO were inoculated with 160 μL of each bacterial suspension (10^7^ CFU/mL). The plates were then incubated 24 h at 30 °C for *A. hydrophila* and *V. parahaemolyticus* and at 37 °C for the other strains. The FIC index was calculated as: ΣFIC = FIC_CEAE_ + FIC_EO_, where FIC_CEAE_ = MIC_CEAE_ combination/MIC_CEAE_ alone and FIC_EO_ = MIC_EO_ combination/MIC_EO_ alone. The interaction results were interpreted as synergy (ΣFIC < 0.5), addition (0.5 ≤ ΣFIC ≤ 1), indifference (1 < ΣFIC ≤ 4), or antagonism (ΣFIC > 4) [21]. All experiments were performed in triplicate. Antibiotics E and VA were also included in the assay with the ratio of concentrations ranging from 0.0625 to 1 μg/mL.

### 4.9. Statistical Analysis

The data were expressed as mean ± standard deviation (SD) of three replicates. The MIC and FIC values of the CEAE and EO were analyzed by one-way analysis of variance (ANOVA), followed by a Fisher’s least significant difference (LSD) at the threshold of *P <* 0.05. 

## 5. Conclusions

This study underlines that the endophytic actinomycete *S. griseorubens* MPT42 associated with medicinal plant *L. cubeba* can be a potential producer of broad-spectrum antimicrobial substances. The cytotoxic effect of *L. cubeba* EO was explored to understand its mode of action on *E. coli* cells. Moreover, the combination of *S. griseorubens* MPT42-extract and EO from the host plant revealed remarkable synergistic antimicrobial effects toward various microbes, and this synergy was equivalent to those between EO and antibiotics (E and VA). In the presence of EO, the MIC of VA was decreased on all Gram-negative bacteria tested and exhibited five additive–synergistic effects. These results underline that the combined antibiotic–EO and CEAE–EO would be an effective strategic choice to reduce the use of antibiotics and fight the emergence and spread of multidrug-resistant microbes. Further studies aim to isolate *S. griseorubens* MPT42-derived antimicrobial substances for determining their mode of action and molecular targets.

## Figures and Tables

**Table 1:** Antibacterial activity of the cell-free supernatant of endophytic actinomycete MPT42 against selected bacteria including pathogens.

Bacterial Species	Zone of Inhibition (D ± SD, mm) ^#^
**Gram-positive bacteria**	
*Listeria innocua* ATCC 33090	27.5^d^ ± 0.5
*Staphylococcus aureus* ATCC 25923	44.0^a^ ± 2.0
*Bacillus cereus* ATCC 13061	18.0^f^ ± 2.0
*Bacillus subtilis* ATCC 11778	36.3^b^ ± 1.1
MRSE	28.2^d^ ± 0.5
**Gram-negative bacteria**	
*Escherichia coli* ATCC 25922	32.3^c^ ± 0.4
*Salmonella* Typhimurium ATCC 14028	24.8^e^ ± 0.9
*Proteus vulgaris* ATCC 49132	11.4^h^ ± 0.4
*Pseudomonas aeruginosa* ATCC 9027	17.2^g^ ± 0.9
*Enterobacter aerogenes* ATCC 13048	18.3^f^ ± 0.4

^#^: Mean of diameter of inhibition zone (D) ± standard deviation (SD) of three replicates. Mean values with different letters (a–h) are significantly different according to the Fisher LCD test (*P* < 0.05).

**Table 2:** Physiological and biochemical characteristics of the actinomycete MPT42.

Characteristic	Result	Characteristic	Result
**Morphological Characteristics**		Fructose	+
Aerial mycelium	Grey and white	Rhamnose	+
Substrate mycelium	Faint-brown	Saccharose	−
Diffusible pigment	−	Sorbitol	+
Spore chain	Spiral	Trehalose	+
Spore surface	Spiny	Asparagin	+
Spore shape	Oval-shaped	Histidine	−
**Physiological Properties**		Phenylalanin	−
Temperature range for growth	25–37 °C	Leucin	+
Optimum temperature	35 °C	Tryptophan	+
pH range for growth	6–10	Arginin	+
Optimum pH	8	Isoleucin	+
NaCl range for growth	0.5–5%	Valin	+
Optimum NaCl	1%	Methionin	+
**Biochemical Properties**		Lysin	+
Glucose	+	Threonin	+
Galactose	+	Cystein	+
Mantose	+	Manitol	+
Lactose	−	**Biosynthetic Genes**	
Arabinose	+	*pks*-I	+
Glucosamine	+	*pks*-II	+
Myo-inositol	+	*nrps*	+

+ Positive; − negative.

**Table 3:** Antimicrobial activity of *S. griseorubens* MPT42-CEAE and *L. cubeba* fruit EO against microbial strains including pathogens.

Bacteria	MIC of Antimicrobial Agents (mean ± SD, µg/mL)			
	EO	CEAE	Erythromycin	Vancomycin
**Gram-positive bacteria**				
*Bacillus cereus* ATCC 13061	2800 ± 0	300 ± 0	6.7 ± 2.3	1.0 ± 0
*Bacillus subtilis* ATCC 11778	2800 ± 0	200 ± 0	10.7 ± 4.6	2.0 ± 0
*Staphylococcus aureus* ATCC 25923	2800 ± 0	80 ± 0	0.7 ± 0.3	1.0 ± 0
MRSA	3700 ± 1.6	1500 ± 0	4.0 ± 0	2.0 ± 0
MRSE	3700 ± 1.6	400 ± 0.1	8.0 ± 0	3.3 ± 1.2
*Listeria innocua* ATCC 33090	1200 ± 0.4	600 ± 0	16.0 ± 0	16.0 ± 0.6
**Gram-negative bacteria**				
*Aeromonas hydrophila* ATCC 35654	2800 ± 0	600 ± 0	128.0 ± 0	128.0 ± 0
*Escherichia coli* ATCC 25922	5500 ± 0	100 ± 0	32.0 ± 0	64.0 ± 0
*Proteus vulgaris* ATCC 49132	700 ± 0	600 ± 0	16.0 ± 0	64.0 ± 0
*Pseudomonas aeruginosa* ATCC 9027	2300 ± 0.8	400 ± 0.1	256.0 ± 0	32.0 ± 0
*Salmonella* Typhimurium ATCC 14028	5500 ± 0	100 ± 0	64.0 ± 0	64.0 ± 0
*Vibrio parahaemolyticus* ATCC 17802	5500 ± 0	200 ± 0	42.7 ± 18.5	128.0 ± 0

**Table 4:** Interaction between *L. cubeba* fruit EO and CEAE, antibiotics expressed as the fractional inhibitory concentration (FIC) index.

Bacterial Species	Combined EO and Antimicrobial Agents					
	CEAE		E		VA	
	MIC (μg/mL)	FIC Index* (mean ± SD)	MIC (μg/mL)	FIC Index * (mean ± SD)	MIC (µg/mL)	FIC Index* (mean ± SD)
**Gram-positive Bacteria**						
*B. cereus* ATCC 13061	20	0.31 ± 0.0 (**S**)	0.56	0.25 ± 0.06 (**S**)	1.0	1.1 ± 0.04 (I)
*B. subtilis* ATCC 11778	50	0.31 ± 0.0 (**S**)	0.89	0.21 ± 0.04 (**S**)	0.21	0.35 ± 0.04 (**S**)
*S. aureus* ATCC 25923	10	0.27 ± 0.04 (**S**)	0.04	0.19 ± 0.00 (**S**)	0.08	0.21 ± 0.04 (**S**)
MRSA ATCC 33591	380	0.58 ± 0.14 (**A**)	0.67	0.23 ± 0.07 (**S**)	0.05	0.50 ± 0.0 (**A**)
MRSE ATCC 35984	50	0.19 ± 0.0 (**S**)	2.67	0.42 ± 0.13 (**S**)	1.65	0.58 ± 0.04 (**A**)
*L. innocua* ATCC 35984	40	0.19 ± 0.0 (**S**)	2.0	0.63 ± 0.11 (**A**)	3.33	0.38 ± 0.0 (**S**)
**Gram-negative Bacteria**						
*A. hydrophila* ATCC 35654	60	0.35 ± 0.04 (**S**)	42.7	0.54 ± 0.07 (**A**)	8.0	0.31 ± 0.0 (**S**)
*E. coli* ATCC 25922	20	0.27 ± 0.04 (**S**)	4.0	0.23 ± 0.04 (**S**)	16.0	0.56 ± 0.0 (**A**)
*P. vulgaris* ATCC 49132	60	0.60 ± 0.04 (**A**)	1.33	0.33 ± 0.04 (**S**)	26.7	0.54 ± 0.04 (**A**)
*P. aeruginosa* ATCC 9027	100	0.31 ± 0.0 (**S**)	21.3	1.08 ± 0.04 (I)	2.67	0.33 ± 0.04 (**S**)
*S.* Typhimurium ATCC 14028	6	1.06 ± 0.0 (I)	32.0	0.56 ± 0.0 (**A**)	53.3	1.83 ± 0.29 (I)
*V. parahaemolyticus* ATCC 17802	100	0.56 ± 0.0 (**A**)	14.2	0.58 ± 0.14 (**A**)	26.7	0.54 ± 0.07 (**A**)

CEAE: crude ethyl acetate extract; E: Erythromycin; VA: Vancomycin. * Synergism (**S**): ΣFIC < 0.5; Additive (**A**): 0.5 ≤ ΣFIC ≤ 1; Indifferent (**I**): 1 < ΣFIC ≤ 4 [20,21]. NA: Not applicable.

## References

[1] Ventola C.L. (2015). The antibiotic resistance crisis: Part 1: Causes and threats. Pharm. Ther..

[2] Alekshun M.N., Levy S.B. (2007). Molecular mechanisms of antibacterial multidrug resistance. Cell.

[3] Yap P.S., Krishnan T., Yiap B.C., Hu C.P., Chan K.G., Lim S.H. (2014). Membrane disruption and anti-quorum sensing effects of synergistic interaction between *Lavandula angustifolia* (lavender oil) in combination with antibiotic against plasmid-conferred multi-drug-resistant *Escherichia coli*. J. Appl. Microbiol..

[4] Wang H., Liu Y. (2010). Chemical composition and antibacterial activity of essential oils from different parts of *Litsea cubeba*. Chem. Biodivers..

[5] Bhuinya T., Singh P., Mukherjee S. (2010). *Litsea cubeba*-Medicinal values-Brief summary. J. Trop. Med. Plants.

[6] Chen Y., Wang Y., Han X., Si L., Wu Q., Lin L. (2013). Biology and chemistry of *Litsea cubeba*, a promising industrial tree in China. J. Essent. Oil. Res..

[7] Nguyen H.V., Caruso D., Lebrun M., Nguyen N.T., Trinh T.T., Meile J.C., Chu-Ky S., Sarter S. (2016). Antibacterial activity of *Litsea cubeba* (*Lauraceae*, *May Chang*) and its effects on the biological response of common carp *Cyprinus carpio* challenged with *Aeromonas hydrophila*. J. Appl. Microbiol..

[8] Nguyen H.V., Vu T.H.N., T.T. V., Phi Q.-T., Khieu T.N., Sarter S., Chu-Ky S. (2016). Antimicrobial activities and interaction effects of Vietnamese *Litsea Cubeba* (lour.) pers essential oils and its endophytic actinobacteria. Vietnam. J. Sci. Technol..

[9] Saikia A.K., Chetia D., D’Arrigo M., Smeriglio A., Strano T., Ruberto G. (2013). Screening of fruit and leaf essential oils of *Litsea cubeba* Pers. from north-east India – chemical composition and antimicrobial activity. J. Essent. Oil. Res..

[10] Wang Y., Jiang Z.-T., Li R. (2012). Antioxidant activity, free radical scavenging potential and chemical composition of *Litsea cubeba* essential oil. J. Essent. Oil. Bear. Plant.

[11] Ho C.L., Jie-Pinge O., Liu Y.C., Hung C.P., Tsai M.C., Liao P.C., Wang E.I., Chen Y.L., Su Y.C. (2010). Compositions and *in vitro* anticancer activities of the leaf and fruit oils of *Litsea cubeba* from Taiwan. Nat. Prod. Commun..

[12] Golinska P., Wypij M., Agarkar G., Rathod D., Dahm H., Rai M. (2015). Endophytic actinobacteria of medicinal plants: Diversity and bioactivity. Antonie van Leeuwenhoek.

[13] Vu T.H.N., Nguyen Q.H., Dinh T.M.L., Quach N.T., Khieu T.N., Hoang H., Chu-Ky S., Vu T.T., Chu H.H., Lee J. (2019). Endophytic actinomycetes associated with *Cinnamomum cassia* Presl in Hoa Binh province, Vietnam: Distribution, antimicrobial activity and, genetic features. J. Gen. Appl. Microbiol..

[14] Da Silva T.F., Vollu R.E., Jurelevicius D., Alviano D.S., Alviano C.S., Blank A.F., Seldin L. (2013). Does the essential oil of *Lippia sidoides* Cham. (pepper-rosmarin) affect its endophytic microbial community?. BMC Microbiol..

[15] Zhou A., Kang T.M., Yuan J., Beppler C., Nguyen C., Mao Z., Nguyen M.Q., Yeh P., Miller J.H. (2015). Synergistic interactions of vancomycin with different antibiotics against Escherichia coli: Trimethoprim and nitrofurantoin display strong synergies with vancomycin against wild-type E. coli. Antimicrob. Agents Chemother..

[16] Lam P.N., Dang T.T.D., Vu T.H.N., Chu-Ky S., Vu T.T., Phi Q.T. (2017). Distribution and antimicrobial activity of endophytic actinomycetes isolated from *Litsea cubeba* (Lour.) Pers. in Northern provinces of Vietnam. Vietnam J. Sci. Technol..

[17] Kuete V. (2010). Potential of Cameroonian plants and derived products against microbial infections: A review. Planta Medica.

[18] Liu T.T., Yang T.S. (2012). Antimicrobial impact of the components of essential oil of *Litsea cubeba* from Taiwan and antimicrobial activity of the oil in food systems. Int. J. Food. Microbiol..

[19] Nguyen H.V., Meile J.C., Lebrun M., Caruso D., Chu-Ky S., Sarter S. (2018). *Litsea cubeba* leaf essential oil from Vietnam: Chemical diversity and its impacts on antibacterial activity. Lett. Appl. Microbiol..

[20] Van Vuuren S., Suliman S., Viljoen A. (2009). The antimicrobial activity of four commercial essential oils in combination with conventional antimicrobials. Lett. Appl. Microbiol..

[21] Gutierrez J., Barry-Ryan C., Bourke P. (2009). Antimicrobial activity of plant essential oils using food model media: Efficacy, synergistic potential and interactions with food components. Food Microbiol..

[22] Christina A., Christapher V., Bhore S.J. (2013). Endophytic bacteria as a source of novel antibiotics: An overview. Pharmacogn. Rev..

[23] Qin S., Li J., Chen H.H., Zhao G.Z., Zhu W.Y., Jiang C.L., Xu L.H., Li W.J. (2009). Isolation, diversity, and antimicrobial activity of rare actinobacteria from medicinal plants of tropical rain forests in Xishuangbanna, China. Appl. Environ. Microbiol..

[24] Al-Askar A.A., Rashad Y.M., Hafez E.E., Abdulkhair W.M., Baka Z.A., Ghoneem K.M. (2015). Characterization of alkaline protease produced by *Streptomyces griseorubens* E44G and its possibility for controlling *Rhizoctonia* root rot disease of corn. Biotechnol. Equip..

[25] Takahashi Y., Nakashima T. (2018). Actinomycetes, an inexhaustible source of naturally occurring antibiotics. Antibiotics.

[26] Bieber B., Nuske J., Ritzau M., Grafe U. (1998). Alnumycin a new naphthoquinone antibiotic produced by an endophytic *Streptomyces* sp.. J. Antibiot. (Tokyo).

[27] Castillo U.F., Strobel G.A., Ford E.J., Hess W.M., Porter H., Jensen J.B., Albert H., Robison R., Condron M.A.M., Teplow D.B. (2002). Munumbicins, wide-spectrum antibiotics produced by *Streptomyces* NRRL 30562, endophytic on Kennedia nigriscansa. Microbiology.

[28] Inahashi Y., Iwatsuki M., Ishiyama A., Namatame M., Nishihara-Tsukashima A., Matsumoto A., Hirose T., Sunazuka T., Yamada H., Otoguro K. (2011). Spoxazomicins A-C, novel antitrypanosomal alkaloids produced by an endophytic actinomycete, *Streptosporangium oxazolinicum* K07-0460(T). J. Antibiot. (Tokyo).

[29] Li W.R., Shi Q.S., Liang Q., Xie X.B., Huang X.M., Chen Y.B. (2014). Antibacterial activity and kinetics of *Litsea cubeba* oil on *Escherichia coli*. PLoS ONE.

[30] Benitez N.P., Meléndez León E.M., Stashenko E.E. (2009). Eugenol and methyl eugenol chemotypes of essential oil of species *Ocimum gratissimum* L. and *Ocimum campechianum* Mill. from Colombia. J. Chromatogr. Sci..

[31] Ooi L.S.M., Li Y., Kam S.-L., Wang H., Wong E.Y.L., Ooi V.E.C. (2006). Antimicrobial activities of cinnamon oil and cinnamaldehyde from the Chinese medicinal herb *Cinnamomum cassia* Blume. Am. J. Chinese Med..

[32] Trinh N.T.T., Dumas E., Thanh M.L., Degraeve P., Amara C.B., Gharsallaoui A., Oulahal N. (2015). Effect of a Vietnamese *Cinnamomum cassia* essential oil and its major component trans-cinnamaldehyde on the cell viability, membrane integrity, membrane fluidity, and proton motive force of *Listeria innocua*. Can. J. Microbiol..

[33] Bouhdid S., Abrini J., Amensour M., Zhiri A., Espuny M.J., Manresa A. (2010). Functional and ultrastructural changes in *Pseudomonas aeruginosa* and *Staphylococcus aureus* cells induced by *Cinnamomum* verum essential oil. J. Appl. Microbiol..

[34] De Sousa J.P., de Azerêdo G.A., de Araújo Torres R., da Silva Vasconcelos M.A., da Conceição M.L., de Souza E.L. (2012). Synergies of carvacrol and 1,8-cineole to inhibit bacteria associated with minimally processed vegetables. Int. J. Food. Microbiol..

[35] Mahboubi M., Ghazian Bidgoli F. (2010). Antistaphylococcal activity of *Zataria multiflora* essential oil and its synergy with vancomycin. Phytomedicine.

[36] Van Vuuren S., Viljoen A. (2011). Plant-based antimicrobial studies-methods and approaches to study the interaction between natural products. Planta. Med..

[37] Langeveld W.T., Veldhuizen E.J., Burt S.A. (2014). Synergy between essential oil components and antibiotics: A review. Crit. Rev. Microbiol..

[38] Ozbek-Celik B., Damar-Celik D., Mataraci-Kara E., Bozkurt-Guzel C., Savage P.B. (2019). Comparative In Vitro Activities of First and Second-Generation Ceragenins Alone and in Combination with Antibiotics Against Multidrug-Resistant *Klebsiella pneumoniae* Strains. Antibiotics.

[39] Mgbeahuruike E.E., Stålnacke M., Vuorela H., Holm Y. (2019). Antimicrobial and Synergistic Effects of Commercial Piperine and Piperlongumine in Combination with Conventional Antimicrobials. Antibiotics.

[40] Adwan G., Mhanna M. (2008). Synergistic effects of plant extracts and antibiotics on Staphylococcus aureus strains isolated from clinical specimens. Middle-East J. Sci. Res..

[41] Yeh P., Tschumi A.I., Kishony R. (2006). Functional classification of drugs by properties of their pairwise interactions. Nat. Genet..

[42] Holder I.A., Boyce S.T. (1994). Agar well diffusion assay testing of bacterial susceptibility to various antimicrobials in concentrations non-toxic for human cells in culture. Burns.

[43] Shirling E.T., Gottlieb D. (1966). Methods for characterization of *Streptomyces* species 1. Int. J. Syst. Evol. Microbiol..

[44] Goodfellow M., Kumar Y. (2010). Reclassification of *Streptomyces hygroscopicus* strains as *Streptomyces aldersoniae* sp. nov., *Streptomyces angustmyceticus* sp. nov., comb. nov., *Streptomyces ascomycinicus* sp. nov., *Streptomyces decoyicus* sp. nov., comb. nov., *Streptomyces milbemycinicus* sp. nov. and *Streptomyces wellingtoniae* sp.. Int. J. Syst. Evol. Microbiol..

[45] Vu H.T., Nguyen D.T., Nguyen H.Q., Chu H.H., Chu S.K., Chau M.V., Phi Q.T. (2018). Antimicrobial and Cytotoxic Properties of Bioactive Metabolites Produced by *Streptomyces cavourensis* YBQ59 Isolated from *Cinnamomum cassia* Prels in Yen Bai Province of Vietnam. Curr. Microbiol..

[46] Phi Q.T., Park Y.M., Seul K.J., Ryu C.M., Park S.H., Kim J.G., Ghim S.Y. (2010). Assessment of root-associated *paenibacillus polymyxa* groups on growth promotion and induced systemic resistance in pepper. J. Microbiol. Biotechnol..

[47] Tamura K., Stecher G., Peterson D., Filipski A., Kumar S. (2013). MEGA6: Molecular evolutionary genetics analysis version 6.0. Mol. Biol. Evol..

[48] Metsa-Ketela M., Salo V., Halo L., Hautala A., Hakala J., Mantsala P., Ylihonko K. (1999). An efficient approach for screening minimal PKS genes from *Streptomyces*. FEMS Microbiol. Lett..

[49] Ayuso-Sacido A., Genilloud O. (2005). New PCR primers for the screening of NRPS and PKS-I systems in actinomycetes: Detection and distribution of these biosynthetic gene sequences in major taxonomic groups. Microb. Ecol..

[50] Salam N., Khieu T.N., Liu M.J., Vu T.T., Chu-Ky S., Quach N.T., Phi Q.T., Narsing Rao M.P., Fontana A., Sarter S. (2017). Endophytic Actinobacteria Associated with *Dracaena cochinchinensis* Lour.: Isolation, Diversity, and Their Cytotoxic Activities. Biomed. Res. Int..

[51] Visvalingam J., Holley R.A. (2012). Temperature-dependent effect of sublethal levels of cinnamaldehyde on viability and morphology of *Escherichia coli*. J. Appl. Microbiol..

